# Mid-term echocardiographic follow up of left ventricular function with permanent right ventricular pacing in pediatric patients with and without structural heart disease

**DOI:** 10.1186/1476-7120-5-13

**Published:** 2007-03-12

**Authors:** Tchavdar Nikolov Shalganov, Dora Paprika, Radu Vatasescu, Attila Kardos, Attila Mihalcz, Laszlo Kornyei, Andras Szatmari, Tamas Szili-Torok

**Affiliations:** 1 Department of Pacing and Clinical Electrophysiology, Gottsegen Gyorgy National Institute of Cardiology, Budapest, Hungary; 2 Center for Pediatric Cardiology, Gottsegen Gyorgy National Institute of Cardiology, Budapest, Hungary

## Abstract

**Background:**

Chronic right ventricular apical pacing may have detrimental effect on left ventricular function and may promote to heart failure in adult patients with left ventricular dysfunction.

**Methods:**

A group of 99 pediatric patients with previously implanted pacemaker was studied retrospectively. Forty-three patients (21 males) had isolated congenital complete or advanced atrioventricular block. The remaining 56 patients (34 males) had pacing indication in the presence of structural heart disease. Thirty-two of them (21 males) had isolated structural heart disease and the remaining 24 (13 males) had complex congenital heart disease. Patients were followed up for an average of 53 ± 41.4 months with 12-lead electrocardiogram and transthoracic echocardiography. Left ventricular shortening fraction was used as a marker of ventricular function. QRS duration was assessed using leads V_5 _or II on standard 12-lead electrocardiogram.

**Results:**

Left ventricular shortening fraction did not change significantly after pacemaker implantation compared to preimplant values overall and in subgroups. In patients with complex congenital heart malformations shortening fraction decreased significantly during the follow up period. (0.45 ± 0.07 vs 0.35 ± 0.06, p = 0.015). The correlation between the change in left ventricular shortening fraction and the mean increase of paced QRS duration was not significant. Six patients developed dilated cardiomyopathy, which was diagnosed 2 months to 9 years after pacemaker implantation.

**Conclusion:**

Chronic right ventricular pacing in pediatric patients with or without structural heart disease does not necessarily result in decline of left ventricular function. In patients with complex congenital heart malformations left ventricular shortening fraction shows significant decrease.

## Background

Chronic right ventricular (RV) apical pacing alters unfavorably left ventricular (LV) electrical activation, mechanical contraction, cardiac output, myocardial perfusion and histology. Permanent RV pacing may have detrimental effect on LV function and may promote to heart failure in adult patients with LV dysfunction [1-10]. The effect of chronic RV apical pacing on LV performance in pediatric patients, although increasingly reported, is not fully investigated yet. Data about this are controversial [5,8,11,12]. Also, although congenital atrioventricular (AV) block quite often is associated with other congenital heart diseases [13-15], studies were mostly focused on patients with isolated congenital AV block [8-16]. The aim of this retrospective study was to assess the evolution of LV function in a mixed pediatric pacemaker population with and without structural heart disease.

## Methods

### Study population

A group of 99 pediatric patients (55 males) was studied. Forty-three patients (21 males) had isolated complete or advanced AV block with severe bradycardia/pauses, syncope, LV dilation or QT interval prolongation. The remaining 56 patients had pacing indication and associated structural heart disease: 23 of them (13 males) had complex congenital heart disease and the remaining 33 (21 males) had isolated structural heart disease (Table 1). The pacing indication in the latter 2 groups was postoperative AV block in 28 patients, congenital AV block in 14 patients, sinus node disease in 10 patients, LV outflow tract obstruction relief in 2 patients, and prolonged QT interval with syncope in 2 patients.

**Table 1:** Isolated structural heart disease and complex congenital heart disease

Isolated structural heart disease/ISHD	33
TGA	5
AVSD	5
ASD	2
AoI	3
ToF	5
PDA	2
VSD + PFO	3
VSD	2
CoAo	3
Sick sinus syndrome + bifurcational pulmonary stenosis	3

Complex congenital heart disease/CCHD	23

VSD, ASD, pulmonary atresia	3
CoAo, VSD, PDA	3
VSD, ASD, TGA, PDA	5
VSD, TGA	4
VSD, PDA, PFO	1
VSD, DORV, PS	2
VSD, DORV, TGA	2
TGA, PS	2
VSD, PDA, pulmonary atresia	1

TGA – transposition of the great arteries; AVSD – atrioventricular septal defect; ASD – atrial septal defect; AoI – aortic insufficiency; ToF – tetralogy of Fallot; PDA – patent ductus arteriosus; VSD – ventricular septal defect; PFO – persistent foramen ovale; CoAo – Aortic coarctation; DORV – double outlet right ventricle; PS – pulmonary stenosis; HOCM – hypertrophic obstructive cardiomyopathy; ARVD – arrhythmogenic right ventricular dysplasia.

All patients had single chamber (right ventricle) or dual chamber (right atrium, right ventricle) systems implanted. Patients with dual-chamber systems pacing only the atrium as well as patients having less than 80% RV pacing (assessed by pacemaker diagnostics) were excluded from the study. Patients were followed up with transthoracic echocardiography and 12-lead electrocardiogram. QRS duration measurements were performed on electrocardiogram with recording speed of 50 mm/sec or 25 mm/sec in lead V_5 _or lead II. Left ventricular shortening fraction defined as: (end-diastolic diameter – end-systolic diameter)/end-diastolic diameter, was used as a marker of LV function. Data on LV function were analyzed in six pediatric age groups (<1 yr; 1–2 yrs; 3–4 yrs; 5–7 yrs; 8–11 yrs and 12–15 yrs).

Comparisons were performed between the last LV shortening fraction value before pacemaker implantation and the LV shortening fraction value at last follow-up after pacemaker implantation, in the whole group and within the 3 subgroups of patients. Comparison was also made throughout the follow up from LV shortening fraction in age groups. Correlation analysis was performed between the mean change of LV shortening fraction and the mean increase of QRS duration before and after pacemaker implantation.

### Statistical analysis

Data are presented as mean ± standard deviation. Statistical analysis was performed using paired and independent samples T-test, and Spearman's ρ correlation. P < 0.05 was considered significant.

## Results

Overall patients were followed up for an average of 53 ± 41.4 months (range 0.5–188.5 months). The mean age at pacemaker implantation in isolated AV block subgroup was 44.1 ± 48 months with a median of 29 months (range 0–139.25). Almost half of those patients had their pacemaker implanted during the first year of life. Patients in this subgroup were followed-up on average for 50.2 ± 42.2 months (range 0.5–188.5).

The mean age at pacemaker implantation in complex congenital heart disease subgroup was 45.4 ± 39.8 months with a median of 38.4 months (range 0–173). The mean follow-up in this subgroup was 58.1 ± 42.2 months (range 3–168). Two patients with complex congenital heart disease died during the follow-up from sudden cardiac death.

The mean age at pacemaker implantation in isolated structural heart disease subgroup was 60.1 ± 53.5 months with a median of 48.6 months (range 0–163). The mean follow-up in this subgroup was 52.8 ± 40.7 months (range 3–152). One patient in this subgroup died during the follow-up from intractable end-stage heart failure.

**Figure 1:**
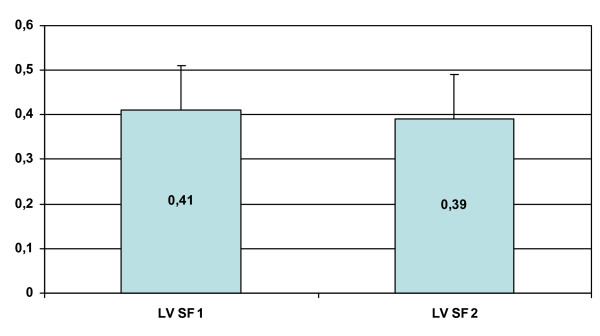
Left ventricular fractional shortening (LV FS) before PM implantation (LV FS 1) and at last follow up (LV FS 2).

Neither the mean age at pacemaker implantation nor the mean duration of the follow-up showed significant difference between the subgroups of patients (p = NS). The mean LV shortening fraction did not change significantly at last follow-up after pacemaker implantation compared to last preimplant values overall and in the subgroups (Table 2 and figure 1).

**Table 2:** Evolution of left ventricular shortening fraction

	Last LV SF before PMI	LV SF at last follow-up	p
Overall	0.41 ± 0.09	0.39 ± 0.11	NS
ICAVB	0.40 ± 0.07	0.37 ± 0.09	NS
CCHD	0.43 ± 0.10	0.39 ± 0.09	NS
ISHD	0.40 ± 0.11	0.41 ± 0.15	NS

CCHD – patients with complex congenital heart disease

ICAVB – patients with isolated congenital complete or advanced AV block

ISHD – patients with isolated structural heart disease

LV SF – left ventricular shortening fraction

NS – not significant

PMI – pacemaker implantation

When analysis was performed throughout the age groups, the change of mean LV shortening fraction was not significant overall and in the subgroups with isolated complete AV block and isolated structural heart disease. However, in the subgroup with complex congenital heart disease a significant decrease of mean LV shortening fraction was observed (patients aged <1 year: 0.45 ± 0.07 versus patients aged 12–15 years: 0.35 ± 0.06, p = 0.015) (Table 3).

**Table 3:** Evolution of left ventricular shortening fraction throughout the age groups.

	LV SF in pts < 1 year	LV SF in pts 12–15 yrs	p
Overall	0.43 ± 0.09	0.4 ± 0.12	NS
ICAVB	0.39 ± 0.08	0.41 ± 0.07	NS
CCHD	0.45 ± 0.07	0.35 ± 0.06	0.015
ISHD	0.49 ± 0.08	0.42 ± 0.18	NS

CCHD – patients with complex congenital heart disease

ICAVB – patients with isolated congenital complete or advanced AV block

ISHD – patients with isolated structural heart disease

LV SF – left ventricular shortening fraction

NS – not significant

The correlation between the mean change of LV shortening fraction after pacemaker implantation and the mean increase of paced QRS duration was not significant (Table 4).

**Table 4:** Spearman's rho correlation between ΔLV SF and increase of paced vs intrinsic QRS duration.

	Mean ΔLV SF (%)	Mean ΔQRS (ms)	ρ	p
Overall	-0.01 ± 0.12	65.9 ± 25.6	-0.175	NS
ICAVB	-0.02 ± 0.1	71.4 ± 21.6	-0.006	NS
CCHD	-0.04 ± 0.12	54.2 ± 30.2	-0.08	NS
ISHD	0.01 ± 0.15	65 ± 26	-0.349	NS

ΔLV SF – change of left ventricular shortening fraction

ΔQRS – change of paced QRS duration after pacemaker implantation vs intrinsic

QRS duration before pacemaker implantation

CCHD – patients with complex congenital heart disease

ICAVB – patients with isolated congenital complete or advanced AV block

ISHD – patients with isolated structural heart disease

NS – not significant

The correlation between the mean change of LV shortening fraction after pacemaker implantation and the pacing duration was not significant as well. This was true for the overall group and for the subgroups as well. The two extremes for Spearman's rho coefficient in this correlation were -0.209 and 0.22 with a non-significant P value.

Five patients with isolated complete AV block and 1 patient (all males) with ostium secundum type atrial septal defect developed dilated cardiomyopathy during the follow-up. Two of them were anti-Ro/SS-A positive (both with isolated complete AV block). The remaining four were not studied for presence of antibodies. All patients with isolated complete AV block had pacemaker implanted at birth, the remaining patient at the age of 20.5 months. Left ventricular shortening fraction was slightly depressed at the time of pacemaker implantation in 2 patients with complete AV block. Dilated cardiomyopathy was diagnosed 2 to 24 months after pacemaker implantation in all patients with isolated complete AV block and 9 years after pacemaker implantation in the patient with atrial septal defect.

## Discussion

The main finding of this study is that RV apical pacing in children with or without structural heart disease during a mid-term follow-up does not cause significant decrease in LV function as represented by the shortening fraction.

Data from trials with adult patients raised concerns about the negative effect of RV apical pacing on LV function [2,3,6,7,9,10]. Due to longer periods of pacing starting not infrequently immediately after birth, these concerns seem to be even more warranting in pediatric patients. Data about the long-term effect of RV apical pacing in children and adolescents are still controversial, some studies supporting the same negative impact observed in adults [4,5,8], while other show opposite results [12]. The present study did not find any significant decrease of LV shortening fraction in this mixed pediatric population and in the subgroups with isolated complete AV block and isolated structural heart disease. However, we were able to find a statistically significant decline in LV function in the subgroup of patients with complex congenital heart disease.

Only approximately a quarter of the patients with structural heart disease had their pacemaker implanted during the first year of life compared to almost half of the patients with isolated complete AV block. This shift of the median age of pacemaker implantation in patients with structural heart disease compared to isolated complete AV block subgroup reflects mainly the time of palliative or totally corrective surgery for congenital heart disease, which frequently causes conduction disturbances necessitating pacemaker therapy.

In this study the mean increase of QRS duration in patients with isolated complete AV block was greater than in patients with structural heart disease. This was not due to more pronounced effect of RV pacing on the QRS duration, but to narrower intrinsic QRS complex in patients with isolated complete AV block compared to patients with structural heart disease. We were not able to find any significant correlation between the mean change of LV shortening fraction and QRS in neither of age groups or in the whole group.

### Limitations of the study

The study is retrospective. The shortening fraction is probably not the best marker for systolic function, but it is the most consistently measured in the study population. Given the fact that segmental wall motion abnormalities in children are extremely rare, one can assume that shortening fraction is reliable enough in this regard.

## Conclusion

Chronic RV apical pacing in pediatric patients with or without structural heart disease does not necessarily result in decline of LV function. In pediatric patients with complex congenital cardiac malformations a significant decrease of LV shortening fraction was observed. The mean change of LV shortening fraction after pacemaker implantation is not related to the increase of paced QRS duration. Progression to dilated cardiomyopathy after pacemaker implantation is observed in 6% in this population. It can develop late after pacemaker implantation.

## List of abbreviations

AV – atrioventricular

LV – left ventricular

RV – right ventricular

## Competing interests

The author(s) declare that they have no competing interests.

## Authors' contributions

TNS participated in the study design, collected the data, performed statistical analysis and drafted the manuscript, DP and RV participated in the study design and collected the data, TSZT designed the study, AK and AM participated in manuscript and table formation, LK and AS as the pediatric cardiologists of all the patients carried out all the echo's of the patients and follow up. All authors read and approved the final manuscript.
